# Facing the COVID-19: A qualitative research on the impact of the pandemic on autism spectrum disorder (ASD)

**DOI:** 10.1192/j.eurpsy.2021.775

**Published:** 2021-08-13

**Authors:** T. Pretel Luque, A. Vilaregut Puigdesens, N. Farriols Hernando, M. Roca Santos, C. Andrés Gárriz

**Affiliations:** Facultat De Psicologia, Ciències De L’educació I L’esport Blanquerna, Universitat Ramon Llull, Barcelona, Spain

**Keywords:** autism spectrum disorder, family, professionals, COVID-19

## Abstract

**Introduction:**

Lockdown resulting from the experienced pandemic has had a great influence on the emotional and social well-being of the general population. Specifically, it is known that those with an Autism Spectrum Disorder (ASD) and their caregivers had to overcome several challenges during this period. Moreover, this situation has influenced the professionals who work in this field.

**Objectives:**

The aim of this study is to describe the impact, the learnings and the challenges that have arisen for the patients with ASD, their families and professionals during the coronavirus outbreak through progenitors’ and professionals’ perceptions.

**Methods:**

A qualitative research design using focus groups was selected to identify and discuss participants’ experiences, beliefs, perceptions and attitudes. The target population consisted on parents with children with ASD and professionals who work with them. Data was collected via two focus groups. A content was made using the program Atlas.ti to determinate the principal categories and themes that describe the COVID-19 impact.

**Results:**

Findings widely describe the problems faced and difficulties experienced by this population during lockdown and after it. As well as the challenges, opportunities and learning that this situation has offered.

**Conclusions:**

Reflections derived from the study manifest the need of thinking about new models of intervention with children with ASD and their families. Greater attention must be paid to parents’ experiences in order to attend to the actual demands of patients and their caregivers contextualized within our current changing situation.

